# In Vitro Gene Conservation Status and the Quality of the Genetic Resources of Native Hungarian Sheep Breeds

**DOI:** 10.3390/vetsci11080337

**Published:** 2024-07-25

**Authors:** Malam Abulbashar Mujitaba, Alexandra Tokár, Eszter Erika Balogh, Viktória Johanna Debnár, Ariuntungalag Javkhlan, Panka Boglárka Vásárhelyi, István Egerszegi, Szabolcs Tamás Nagy, Gabriella Kútvölgyi

**Affiliations:** 1 Department of Animal Nutrition and Physiology, Faculty of Agriculture and Food Sciences and Environmental Management, University of Debrecen, Böszörményi Street 138, H-4032 Debrecen, Hungary; malam.abulbashar@agr.unideb.hu; 2 Doctoral School of Animal Science, University of Debrecen, Böszörményi Street 138, H-4032 Debrecen, Hungary; 3 Department of Precision Livestock Farming and Animal Biotechnics, Institute of Animal Sciences, Hungarian University of Agriculture and Life Sciences, Kaposvár Campus, Guba Sándor Street 40, H-7400 Kaposvár, Hungary; tokar.alexandra@uni-mate.hu (A.T.); balogh.eszter.erika@uni-mate.hu (E.E.B.); debnar.viktoria.johanna@uni-mate.hu (V.J.D.); kutvolgyi.gabriella@uni-mate.hu (G.K.); 4 Festetics György Doctoral School, Hungarian University of Agriculture and Life Sciences, Deák Ferenc Street 16, H-8360 Keszthely, Hungary; ariunaa0731@gmail.com; 5 Agricultural Engineering Master Student Programme, Institute of Agronomy, Hungarian University of Agriculture and Life Sciences, Szent István Campus, Páter Károly u. 1, H-2100 Gödöllő, Hungary; vasarhelyi.panka.boglarka@stud.uni-mate.hu; 6 Department of Animal Husbandry and Animal Welfare, Institute of Animal Sciences Hungarian University of Agriculture and Life Sciences, Szent István Campus, Páter Károly u. 1, H-2100 Gödöllő, Hungary; egerszegi.istvan@uni-mate.hu; 7 Department of Precision Livestock Farming and Animal Biotechnics, Institute of Animal Sciences, Hungarian University of Agriculture and Life Sciences, Georgikon Campus, Deak F. u. 16, H-8360 Keszthely, Hungary

**Keywords:** Gene Bank, Racka, Tsigai, Cikta, spermatozoa, cryopreservation

## Abstract

**Simple Summary:**

Considering the global decline in local sheep genetic resources, we conducted the current study to assess the status and quality of Hungarian native sheep breed genetic resources stored in a small Gene Bank established between 2014 and 2017. Semen samples from 24 rams of different breeds were collected during the breeding season and out of season using an artificial vagina, cryopreserved manually. Frozen/thawed spermatozoa were evaluated for motility and kinematic parameters, viability, chromatin structure, and morphometry of the sperm nuclei. We observed breed, season, and individual ram effects on the motility, kinematics, viability, and morphometrical parameters of the post-thaw spermatozoa samples stored in the Gene Bank. The stored samples are of good quality, and the cryopreservation technique adopted for the small Gene Bank proved promising for conserving the genetic resources of the native Hungarian sheep, as it resulted in acceptable post-thaw parameters. To maintain rare and valuable genotypes, cryo-conservation of not only spermatozoa but also oocytes and embryos is needed. Therefore, we plan to implement an extended gene conservation program involving a more significant number of individuals of native Hungarian sheep breeds.

**Abstract:**

Studies revealed a global loss of genetic resources for local sheep breeds. Therefore, the current study aimed to introduce and highlight the progress made on Hungary’s existing gene conservation program (small Gene Bank). Furthermore, we evaluated breed (Tsigai, Cikta, and Racka), season, and individual variabilities (n = 24) of the pre-freeze and post-thaw semen stored in the Gene Bank to enhance the gene conservation of the breeds. The samples were cryopreserved manually, and post-thaw spermatozoa were analyzed for motility (CASA), viability, chromatin structure, and morphometry of the sperm nuclei. Ejaculate volume, spermatozoa concentration, subjective motility and standard motility, kinematic parameters, and spermatozoa’s head area standard deviation of the post-thaw samples differed significantly among breeds (*p* < 0.05). Season affected ejaculate volume, total spermatozoa number/ejaculate, STR, BCF, and ALH. We observed a significant (*p* < 0.001; 0.05) breed and season interaction on concentration, total spermatozoa number/ejaculate, VCL, LIN, WOB, spermatozoa’s head average perimeter and nucleus length (Tsigai and Cikta differed but were statistically the same as Racka). Similarly, season significantly (*p* < 0.05) affected the proportion of ejaculate suitable for freezing. There was a significant (*p* < 0.05) difference in kinematic parameters and viability among the rams across the breeds. The spermatozoa’s head morphometry of the Tsigai and Cikta breeds differed significantly (*p* < 0.05) among the rams. There were individual and breed differences in many spermatozoa quality parameters. The stored samples are of good quality, with more than 40% having intact membranes and low abnormal chromatin condensation.

## 1. Introduction

Local sheep breeds are thrifty, hardy, and more adaptable to harsh weather conditions than the improved genotypes [1]. There are many different local sheep breeds in Hungary, such as Hortobágyi Racka (black or white variants), Gyimesi Racka, Cikta, Cigája (Tsigai in English), and Milking Tsigai, forming the heritage breeds [2]. Hungarian Merino and its crossbred are the most reared local sheep breeds [3]. However, most of them are seasonal, with the autumn season being the most favorable, as indicated by the highest serum testosterone levels and ejaculate volume with a lower percentage of spermatozoa abnormality, as compared to the winter season, which recorded the lowest values and a higher percentage of abnormal spermatozoa in Hungarian Racka rams [4]. Furthermore, out of the Hungarian local sheep breeds mentioned earlier, the Tsigai was the most resilient, thrifty, and adaptable to the weather conditions in Hungary [2,3]. The Tsigai (black or coffee-colored on the head and extremities) is a triple-purpose breed (mutton, milk, and wool) originating specifically from Asia Minor and was introduced to Hungary in the 18th century from the Balkans and other neighboring countries [2,5]. The Hungarian Racka sheep breed (black, white, and Transylvanian) originated from the Hungarian lowlands and was threatened by the Merino sheep breed, replacing it in the 19th century [5]. The breeds are vital due to their high fertility, robustness, and adaptability. They are also used in the Pannonia area for landscape conservation of extensively cultivated pastures [5]. The Racka was known for good-quality fleece (adult rams and ewes producing 3–4 kg and 2–3 kg, respectively) and reasonable milk production (40 L per lactation). Its mutton was characterized by less intramuscular fat and higher nutritional value than modern breeds like Merinos, thus giving it a higher consumer preference in the European market in the past [6]. Cikta originated from Zaupel sheep (Zaupelschaf), also called Hungarian Zaupel, Zibbe, and Swabian sheep, which the Swabian settlers introduced to southern Hungary in 1720. The Cikta is a non-seasonal breed, which lambs twice a year, has good milk production (50–60 L per lactation), and has the best-quality wool among the local Hungarian sheep breeds aside from being tolerant to weather extremes [6]. For clear pictures of the different breeds, refer to [7]. 

However, a shift in the demand or a change in the price of a particular animal commodity (*meat, milk, and/or wool*) is the primary driving force for breed modification, which consequently leads to the loss of valuable and adaptable livestock genetic resources, with crossbreeding for meat rendering 50% of certain sheep breeds endangered or vulnerable in the Alpine regions of France and Italy [8]. Similarly, increased selection pressure using assisted reproductive technologies leads to the erosion of valuable local sheep breed genetic resources [9,10]. As of 2021/2022, based on the total number of adult female population, the local risk status of certain Hungarian sheep breeds stands as follows. The Hungarian Polled Racka breed was categorized as critical (33 head) [11], the Yellow Head Berke (328 head) as critical [12], the Milking Tsigai (328 head) as critical [13], the Cikta (681 head) as endangered [14], and the Gyimes Racka (1146 head) as endangered [15], while the Hungarian Black Racka (2632 head), Hungarian White Racka (2703 head), and Tsigai (3495 head) are categorized as vulnerable [16,17,18]. 

Interestingly, there exists a conservation program for local sheep breeds in Hungary supported by Tenders titled of “In situ preservation of the genetic stock of protected native and endangered agricultural animal breeds” [19] and “Ex situ, or in vitro preservation of the genetic stock of protected native and endangered agricultural animal breeds, and support for advisory activities preventing genetic narrowing” [20]. There are 33 Hungarian Polled Racka ewes with 114% prolificacy bred by three breeders and registered in the herd book in 2022. The Tsigai breed seems quite stable due to its population, which reached 3495 heads in 2022 and reared on 52 farms. The genetic resource conservation program of the Hungarian and Transylvanian Racka breed also exists, which led to its restoration in 1983 by the Hungarian Racka Sheep Breeder Association [5]. It is, therefore, imperative to conserve the genetic resources of this valuable local sheep breed. The population of these breeds declined typically due to the shift in the breeders’ interest in rearing/keeping more productive/modern breeds [6]. 

The maintenance, sustainable utilization, and re-establishment of the lost genotypes/biodiversity is referred to as biodiversity/gene conservation and is effectively achieved using in situ or ex situ conservation techniques or their combinations (*ex situ–in situ continuum*), as suggested by IUCN [21]. The in situ conservation technique entails the maintenance of viable populations in their habitat, while the ex situ technique conserves genetic diversity in the zoo, aquarium, or Gene Banks [22,23]. The ex situ technique is further divided into ex situ in vivo (*conserving genetic diversity in a zoo or aquarium*) and ex situ in vitro (*preserving genetic materials at sub-zero temperature, −196 °C*) [9,23]. However, the latter technique receives more attention than the former because it permits indefinite conservation of genetic resources like live spermatozoa, oocytes, embryos, DNA, and chromosomes and provides an opportunity for research and species/breed recovery programs [22]. 

Evaluating breed and individual variabilities of the post-thaw semen quality and comparative studies are essential for planning gene conservation programs and establishing Gene Banks. The current study aimed to introduce and highlight the progress made on the existing gene conservation program (small Gene Bank), which was established in 2014–2017, to evaluate the breed and seasonal effect on seminal quality parameters in fresh ejaculates and post-thaw semen samples collected over this period, and to compare the quality of representative semen batches to determine the future usability of frozen-stored spermatozoa samples in the Gene Bank.

## 2. Materials and Methods 

### 2.1. Ex Situ In Vitro Gene Conservation Program

Ex situ gene conservation programs are supported in Hungary by the tender entitled “Ex situ, or in vitro preservation of the genetic stock of protected native and endangered agricultural animal breeds, and support for advisory activities preventing genetic narrowing” (Tender code number VP4-10.2.1.2-17), announced every 3 years [20]. The source of the support is provided in co-financing by the European Agricultural Rural Development Fund and the budget of Hungary. The purpose of the supported program is to collect genetic material (sperm, embryos, or oocytes) of protected indigenous and endangered agricultural animal breeds and prepare them for freezing procedures, cryopreservation, and deep-frozen storage under laboratory conditions. The Hungarian Sheep and Goat Breeders’ Association applied for the grant in cooperation with the National Agricultural Research and Innovation Center—Research Institute for Animal Breeding, Nutrition and Meat Science, Herceghalom, 2014. During the program, ejaculates of breeding rams of the following native Hungarian sheep breeds were frozen, and a sperm bank was established: Hungarian Racka, Cikta, Tsigai, and Milking Tsigai.

### 2.2. Study Location, Animals

The study was performed at the National Agricultural Research and Innovation Center, Research Institute for Animal Breeding, Nutrition and Meat Science, Herceghalom, Hungary, a former Institute that is currently connected to the Hungarian University of Agriculture and Life Sciences (northern latitude: 47°29′, eastern longitude: 18°44′) between 2014 and 2017.

Altogether, 78 breeding rams were selected by the Hungarian Sheep and Goat Breeders’ Association for the Gene Conservation Program and transported from different farms in Hungary to the Experimental Farm and Artificial Insemination Station of the Research Institute between September 2014 and April 2017. Three or four breeding rams were housed together in pens in the sheepfold. They were fed grass, hay, and concentrate and had access to water ad libitum. The breeding farms of origin of the rams ranged from extensive grazing systems to small family farms. The relationship of animals to humans and trainability was also different. Some of the rams arrived only at the end of the breeding season after being taken out from the harem or during the out of season (OS). The breeding season (BS) for these sheep breeds lasts from August to the end of December. Only one-third of the rams could be trained for semen collection by artificial vagina (AV). Most Racka rams were shy or aggressive towards people because of the extensive keeping system and rare human contact. After an unsuccessful training period, these rams were excluded from the program. Altogether, 24 rams (6 Hortobágyi Racka, 7 Cikta, 8 Tsigai, 3 Milking Tsigai) were involved successfully in the gene conservation program.

### 2.3. Semen Collection, Processing, and Cryopreservation

In the first 30 days of quarantine, veterinary examinations and blood samples for serological tests of Brucella ovis, Brucella melitensis, Border disease, and Bluetongue disease were taken, and the technician started getting the rams used to people. After the quarantine period, rams were trained for semen collection by AV. At the beginning of the sperm collection period, the semen sample of each ram was cultured for bacteriology by the National Food Chain Safety Office, Hungary. Only rams that produced ejaculates free from pathogenic bacteria were used for further semen collection and cryopreservation for the Gene Bank. In the case of any pathogenic bacteria growing in the ejaculate, the ram was treated with antibiotics based on antibiotic susceptibility testing, and then the sampling and bacteriology culture was repeated.

Semen was collected with an AV (IMV technologies, L’Aigle, France) in the presence of an estrous ewe once or twice a week until 200 doses of frozen sperm were produced (Table 1). One or two ejaculates were collected per day with a 15–30 min interval between collections. Ejaculates were stored at 37 °C until and during the semen analysis. Ejaculate volume was determined, and spermatozoa concentration was assessed with a spectrophotometer (Accucell, IMV technologies, L’Aigle, France). Then, the total number of spermatozoa per ejaculate was calculated. The mass movement of sperm cells in undiluted semen was evaluated and scored (0–5) using a phase contrast microscopy (Olympus BX-51, Olympus Life and Material Science Europa GmbH, Hamburg, Germany) at 50× magnification. An aliquot was diluted with Andromed^®^ semen extender (Minitube, Tiefenbach, Germany) at 1:50, at a 1:100 dilution rate depending on sperm concentration of the ejaculate, and spermatozoa motility was subjectively evaluated under a phase contrast microscope at 200× magnification. Semen smears were prepared for viability and morphology evaluation of spermatozoa. At the beginning of the sperm cryopreservation period of each ram and later in the case of visibly defective spermatozoa of the ejaculate, the ratio of morphologically abnormal sperm cells of the raw ejaculate was determined using bright field microscopy at 1000× magnification, which had a limitation of 15% of total spermatozoa. No rams, only some ejaculates, were excluded based on this criteria. The ejaculates, which have more than 2 billion cells per mL sperm concentration and contain more than 75% motile spermatozoa, were further processed. If both ejaculates of the ram were suitable, they were pooled. All data of the collected ejaculates were recorded in a database.

Each semen sample was diluted to a final concentration of 300 × 10^6^ motile spermatozoa/mL with an Andromed^®^ semen extender at room temperature. The extender was prepared according to the manufacturer’s guidelines from the stock solution in one step, then filled into sterilized 10 mL centrifuge tubes, and stored under frozen conditions until required. Diluted semen was manually filled into preprinted differently colored transparent 0.5 mL semen straws and sealed using PVA (IMV technologies, L’Aigle, France). The straws were placed on a freezing rack and equilibrated at 5 °C for 2 h. Freezing was performed manually in a Styrofoam box at 4 cm above the liquid nitrogen (LN_2_) for 8 min; then, frozen straws were plunged into LN_2_. Semen straws were placed into well-labelled goblets for permanent storage in the Gene Bank. After more than two days of storage, one randomly taken straw of each frozen sample was thawed (37 °C for 30 s) and assessed for standard motility and kinematic parameters, and smears were prepared for membrane integrity evaluation. 

### 2.4. Evaluation of Frozen/Thawed Spermatozoa

Motility analysis of all frozen/thawed samples stored in Gene Bank was performed. Three representative frozen semen samples of each ram were selected (from the beginning, the middle, and the end of the semen collection period of each ram) for further analyses. Assessmment of viability, Feulgen fragmentation, and sperm head morphometry was performed for each representative sample.

#### 2.4.1. Motility Assessment

The motility and kinematic parameters of the frozen/thawed spermatozoa were assessed using a Computer-Assisted Sperm Analyzer (CASA) (Spermvision^®^ software, Minitube Ltd., Tiefenbach, Germany). The samples were diluted to a 50–60 × 10^6^ spermatozoa/mL concentration using a preheated Andromed^®^ semen extender (Minitube Ltd., Tiefenbach, Germany). For sperm motility analysis, the Makler chamber (Sefi Medical Instruments, Haifa, Israel) was used under a phase contrast microscope at 200× magnification.

At least 10 random fields per sample, or a total of 500 spermatozoa, were analyzed for total motility (TM, %), progressive motility (PM, %), and kinematic parameters: curvilinear velocity (VCL, μm/s), average path velocity (VAP, μm/s), straight line velocity (VSL, μm/s), linearity (LIN = VSL/VCL), straightness (STR = VSL/VAP), beat cross frequency (BCF, Hz), amplitude of lateral head displacement (ALH, μm), and wobble (WOB = VAP/VCL). Samples with a minimum of 40% total motility were considered acceptable for further long-term storage in the Gene Bank.

#### 2.4.2. Viability Assessment 

**Figure 1:**
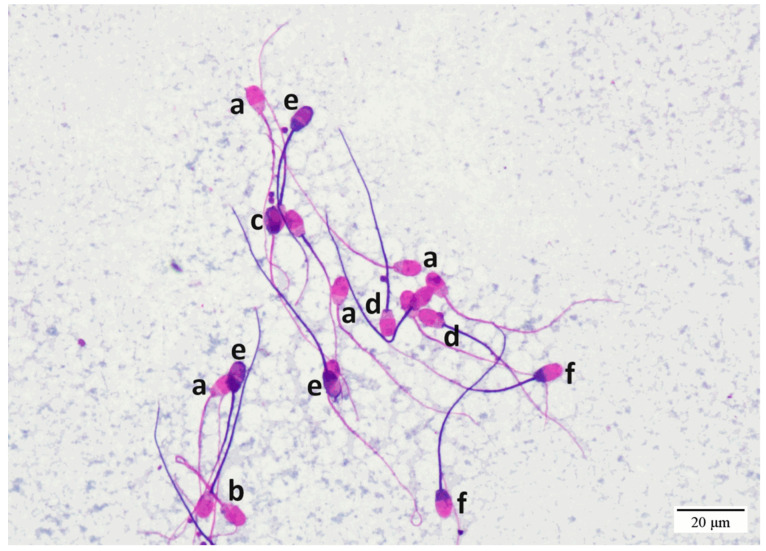
Different post-thaw ram spermatozoa categories stained with the modified Kovács–Foote staining technique (magnification ×400, brightfield optics). (a) Intact head, intact tail, and acrosome membrane (intact: IHITIA). (b) Intact with a bent tail (IBT). (c) Damaged head with intact tail (DHIT). (d) Intact head, damaged tail (IHDT). (e) Damaged head, damaged tail, and damaged acrosome (DHDTDA). (f) Damaged head, damaged tail, and intact acrosome.

Sperm head, tail, and acrosome membrane integrity and morphometry were evaluated by modified Kovács–Foote staining method using 0.16% Chicago sky blue 6B (Sigma-Aldrich, St. Louis, MO, USA, C-8679) viability stain, neutral red (Sigma N 2880) formaldehyde fixation, and Giemsa (Sigma GS-500) acrosome staining, as described in [24,25]. Stained slides were evaluated using an immersion objective with bright field microscopy at 1000× magnification. Three hundred sperm cells were evaluated on one of the two slides prepared and classified into eight categories based on membrane integrity and morphology of the spermatozoa: intact head, intact tail, and acrosome membrane; normal morphology (IHITIA); intact head, tail, and acrosome membrane with a proximal cytoplasmic droplet (IPD); intact head, tail, and acrosome membrane with a distal cytoplasmic droplet (IDD); intact head, tail, and acrosome membrane with a tail defect (bent, curved, broken midpiece or tail, hairpin curved, coiled tail) (IBT); intact head, tail, damaged acrosome membrane (IHITDA); damaged head, intact tail membrane (DHIT); intact head, damaged tail membrane (IHDT); and damaged head and tail membrane with intact or damaged acrosome (DHDT) (Figure 1). The percentages of IHITIA and all spermatozoa with intact entire cell membranes (IHITIA + IPD + IDD + IBT) were calculated in representative frozen samples of each ram. Different spermatozoa categories are shown in Figure 1.

#### 2.4.3. Feulgen Staining 

**Figure 2:**
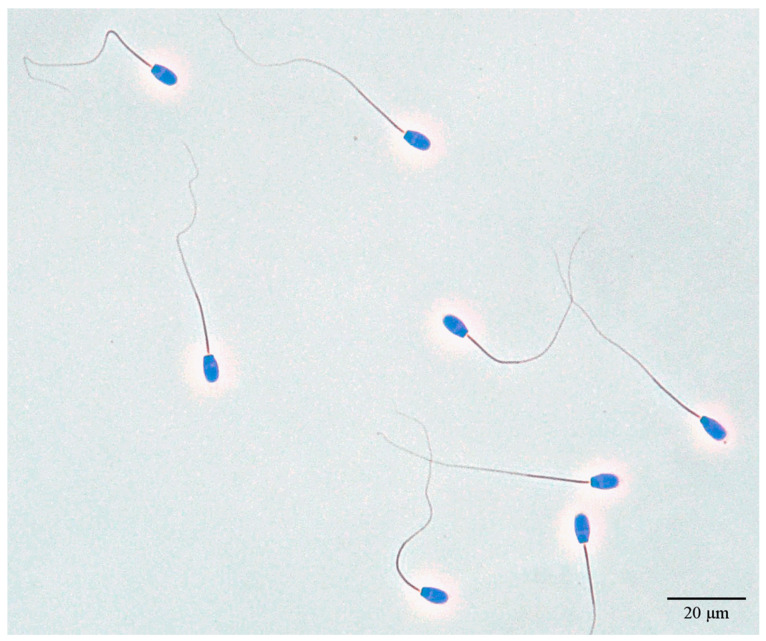
Feulgen-stained ram spermatozoa with intact chromatin structure. Phase contrast optics.

Chromatin condensation and sperm head morphometry analyses were carried out on Feulgen-stained smears of each of the representative frozen semen samples. This procedure allows the measurement of the dimensions of the sperm nucleus without the confounding effect of plasma membrane or acrosome status [26]. We followed the staining protocol provided by the manufacturer of the Feulgen staining kit (Merck KGaA, Darmstadt, Germany, cat. no. 1.07907.0001), with modifications suggested by [27]. Air-dried semen smears were kept in Reagent 1 (5 mol/L hydrochloric acids) at room temperature for 30 min, then washed in running tap water for five minutes. Slides were stained with Reagent 2 (Schiff’s reagent) at room temperature for 30 min, in the dark, then rinsed in sodium disulfite rinsing solution (freshly prepared before use, by adding 1 mL of Reagent 1, 5 mol/L hydrochloric acid, and 5 mL of Reagent 3, concentrated sodium disulfite solution to 95 mL distilled water) for three minutes. This washing step was repeated, and then slides were washed with running water for 10 min. After drying, smears were coverslipped with Entellan (Merck KGaA, Darmstadt, Germany, cat. no. 1.07960). Using phase contrast optics, sperm nuclei with intact chromatin structure show a uniform bluish color (Figure 2), and a dotted pattern indicates abnormal chromatin condensation.

#### 2.4.4. Digital Photography, Image Analysis

Feulgen-stained smears were photographed under a phase contrast microscope (Olympus BX-51, Olympus Life and Material Science Europa GmbH, Hamburg, Germany), using 40× objective with cellSense Standard Imaging Software by Olympus. Digital photos were used to measure the morphometry of the sperm nuclei with ImageJ (ImageJ 1.54g; Java 1.8.0_345 [64-bit]; Windows 10). ImageJ is a free software tool (https://imagej.net/ij/index.html, accessed on 4 July 2024) that measures the size of the area and the perimeter of the sperm nuclei. A total of 200 sperm cells were measured from each ram sample, simultaneously evaluating the chromatin status. Pixel-to-µm ratios were established by photographing a Makler chamber with the same microscope settings. Results were collected into .txt files then transformed to .xls with Microsoft Excel^®^ version 29. Additional analyses were performed on the same digital photos with Sperm Sizer [28], version 1.6.6, to measure sperm nucleus length.

#### 2.4.5. Data Analysis

Fresh and post-thaw semen quality data were collected, recorded, and analyzed for descriptive statistics using IBM^®^ SPSS^®^ statistical software version 29. Normality was checked using a Shapiro–Wilk test, and transformations were achieved using a two-step transformation. A general linear model using Two-way Analysis of Variance (ANOVA) was used to analyze the effects of breed (Tsigai, Cikta, and Racka) and season (breeding season vs. out of season) and their interaction on the basic sperm parameters (volume, total sperm number/ejaculate, mass motility, and subjective motility), standard motility, kinematic parameters, and viability, morphometrical, and chromatin de-condensation parameters with the level of the significance set at *p* < 0.05. The freezability and morphology of the representative ram samples of each breed were analyzed using one-way ANOVA. Means were separated using the Tukey post hoc test. Effects of breeds and season on the proportion of ejaculate freezability based on 75% subjective motility and storage rate in the Gene Bank based on 40% post-thaw TM were analyzed using Chi-square, and the significance difference was checked using a two-tailed test. Results were presented as means ± standard error of means (SE).

## 3. Results

Table 2 presents the main effects of breed and season on the fresh ejaculate quality characteristics of native Hungarian sheep breeds. The spermatozoa volume, concentration, and subjective motility were significantly (*p* < 0.05) affected by breed, while season affected the volume and total spermatozoa number per ejaculate. The Tsigai breed presented significantly (*p* < 0.05) higher spermatozoa volume (0.96 ± 0.02 mL) than the Cikta and Racka breeds, which were statistically the same (0.70 ± 0.03 and 0.79 ± 0.04 mL). Moreover, the Racka breed had significantly (*p* < 0.05) higher spermatozoa concentration than Tsigai and Cikta, while Cikta was significantly (*p* < 0.05) higher than Tsigai. The Racka breed also had significantly (*p* < 0.05) higher subjective motility than the Tsigai and Cikta breeds, which were statistically the same. The spermatozoa volume and the total sperm number/ejaculate were observed to be significantly (*p* < 0.05) higher in the breeding season than out of season, at 0.91 ± 0.03 vs. 0.79 ± 0.02 mL and 3966.80 ± 198.38 vs. 3564.10 ± 195.91 × 10^6^/mL, respectively. The spermatozoa concentration and total sperm number/ejaculate presented significant (*p* < 0.05) breed and season interactions. 

The results of the interaction effect of breed and season on spermatozoa concentration, as well as the total spermatozoa number/ejaculate, are presented in Table 3. The Racka breeds significantly (*p* < 0.001) had the highest value of spermatozoa concentration (5630.79 ± 178.57 × 10^6^/mL), while the Tsigai breed had the lowest during the breeding season (3191.38 ± 174.24 × 10^6^/mL). However, during the out of season, the Cikta had significantly (*p* < 0.05) higher spermatozoa concentrations than the Tsigai (4750.33 ± 254.57 vs. 3937.81 ± 183.70 × 10^6^/mL), while the Racka breed had similar values (4639.24 ± 273.97 × 10^6^/mL) compared to Tsigai and Cikta rams. The Tsigai had significantly higher spermatozoa concentration during the out of season than the breeding season (3937.81 ± 183.70 vs. 3191.38 ± 174.24 × 10^6^/mL). In contrast, the Racka breed had a substantially higher concentration during the breeding than out of season (5630.79 ± 178.57 vs. 4639.24 ± 273.97 × 10^6^/mL). The total sperm number/ejaculate was significantly different (*p* < 0.001) among the breeds during the breeding season, with the Racka breed presenting the highest value (5185.53 ± 485.88 × 10^6^/mL) compared to the Tsigai and Cikta breeds, which did not differ statistically, at 3478.92 ± 238.69 and 3411.79 ± 278.30 × 10^6^/mL, respectively. The Racka breed had a significantly (*p* < 0.001) higher total sperm number/ejaculate during the breeding season than out of season (5185.53 ± 485.88 vs. 3064.66 ± 362.22 × 10^6^/mL). All the Cikta rams’ fresh spermatozoa quality parameters were statistically similar (*p* > 0.05) between the seasons. 

Table 4 presents the effects of breed and season on the proportion of the fresh spermatozoa samples suitable for freezing based on 75% subjective motility and 2 billion/mL spermatozoa concentration threshold values and post-thaw samples ideal for storage in the Gene Bank based on 40.00% total motility of the three native Hungarian sheep breeds. Based on the above-mentioned criteria, there was no significant difference (*p* > 0.05) among the breeds in the proportion of spermatozoa samples frozen and stored in the Gene Bank. Season does not significantly affect (*p* > 0.05) the proportion of stored samples in the Gene Bank. However, it significantly (*p* < 0.05) influenced the proportion of spermatozoa samples frozen and discarded. The out of season resulted in significantly higher percentages of frozen and lower discarded samples than the breeding season, at 66.80 vs. 56.00% and 33.20 vs. 44.00%, respectively. 

Table 5 presents the effects of breed and season on the post-thaw spermatozoa motility and kinematic parameters of some native Hungarian sheep breeds. The Racka breed had significantly (*p* < 0.001) higher TM, PM, and ALH (70.20 ± 1.5%, 65.99 ± 1.7%, and 6.01 ± 0.1 μm) than the Tsigai (60.55 ± 1.1%, 53.92 ± 1.2%, and 5.50 ± 0.1 μm) and Cikta (57.33 ± 1.4, 51.24 ± 1.5%, and 5.65 ± 0.1 μm) breeds. The Cikta and Racka breeds were statistically the same and had significantly (*p* < 0.001) higher curvilinear (203.08 ± 3.3 vs. 209.62 ± 3.7 μm/s), average path (102.18 ± 1.4 vs. 102.77 ± 1.6 μm/s), and straight-line velocity (82.59 ± 1.2 vs. 80.63 ± 1.3 μm/s) than the Tsigai breed, 188.61 ± 2.7, 95.71 ± 1.2, 76.64 ± 1.0 μm/s, respectively. In contrast, the Tsigai and Cikta breeds were statistically the same and had significantly (*p* < 0.001) higher LIN (40.48 ± 0.4 vs. 40.45 ± 0.5%), STR (79.34 ± 0.7 vs. 80.63 ± 0.9%), and WOB (50.72 ± 0.4 vs. 49.85 ± 0.5%) than the Racka breed, 37.98 ± 0.5, 76.54 ± 1.0, 47.64 ± 0.6%, respectively. Samples collected and frozen during out of season presented significantly (*p* < 0.05) higher STR (80.18 ± 0.7 vs. 77.49 ± 0.7%), BCF (32.04 ± 0.3 vs. 31.03 ± 0.3 Hz), and lower ALH (5.60 ± 0.1 vs. 5.84 ± 0.1 μm) than during the breeding season. The VCL, LIN, and WOB presented significant (*p* < 0.05) breed and season interaction.

Table 6 presents the interaction effects of breed and season on post-thaw sperm kinematic parameters of native Hungarian sheep breeds. The Racka and Cikta breeds were statistically similar and had significantly (*p* < 0.001) higher VCL (211.39 ± 3.9 and 203.88 ± 4.0 μm/s) than the Tsigai breed (178.70 ± 3.6 μm/s) during the breeding season. The Tsigai breed VCL was significantly higher (*p* < 0.001) during the out of season than the breeding season (198.53 ± 4.0 vs. 178.70 ± 3.6 μm/s). On the other hand, the Tsigai and Cikta breeds were statistically comparable and had higher LIN (41.12 ± 0.8 and 40.04 ± 0.6%) during the breeding season than the Racka (37.00 ± 0.5%). The Racka post-thaw spermatozoa were significantly (*p* < 0.05) more linear during the out of season than in the breeding season (38.79 ± 0.5 vs. 37.00 ± 0.5%). The Tsigai breed had a significantly (*p* < 0.01) higher WOB parameter (52.30 ± 0.7%) during the breeding season than the Cikta (49.70 ± 0.8%), which was also higher than the Racka breed (47.04 ± 0.8%). The Tsigai breed presented significantly higher (*p* < 0.001) WOB during the breeding season than the out of season (52.30 ± 0.7 vs. 49.15 ± 0.5%). 

Table 7 presents the effects of breed and season on sperm quality parameters of representative post-thaw sperm samples of three native Hungarian ram breeds. The Racka breed presented significantly (*p* < 0.005) higher TM and PM (72.26 ± 2.03 and 67.11 ± 2.26%) than the Tsigai and Cikta breeds, which were statistically the same (62.71 ± 1.73 vs. 55.82 ± 2.07% and 62.86 ± 2.66 vs. 57.29 ± 2.53%). The Racka and Cikta breeds were statistically the same and had significantly (*p* < 0.01) higher VCL than the Tsigai breed, 205.79 ± 5.29 and 205.67 ± 5.73 vs. 186.30 ± 4.41 μm/s, respectively. The Cikta had (*p* < 0.05) significantly higher VAP and VSL than the Tsigai breed (104.112.11 and 84.062.06 μm/s vs. 94.98 ± 1.78 and 76.76 ± 1.41 μm/s), but this was statistically the same as the Racka breed, 100.46 ± 2.59 and 78.49 ± 2.60 μm/s. The Tsigai and Cikta breeds were statistically the same and had significantly (*p* < 0.01) higher LIN, 41.15 ± 0.75 and 40.66 ± 1.08%, than the Racka breed, 37.26 ± 0.64%. The Racka spermatozoa had significantly (*p* < 0.05) higher ALH and lower WOB values than the Tsigai, 6.03 ± 0.09 μm and 48.26 ± 0.51% vs. 5.39 ± 0.13 μm and 50.73 ± 0.53%, respectively, but was statistically the same as Cikta, 5.68 ± 0.17 μm and 50.33 ± 0.77%. The season had no significant effect on all the motility and kinematic parameters of the representative rams’ spermatozoa. However, VCL, LIN, and WOB were the only parameters that presented significant (*p* < 0.05) breed and season interaction.

Table 8 presents the effects of breed and season on some kinematic parameters of representative frozen/thawed sperm samples of the three native Hungarian ram breeds. The Racka and Cikta breeds were statistically the same and had significantly (*p* < 0.001) higher VCL (200.60 ± 9.78 and 202.10 ± 11.70 μm/s) than the Tsigai breed (194.50 ± 10.90 μm/s) during the breeding season. Racka spermatozoa had significantly (*p* < 0.001) lower LIN values than the Tsigai, 39.00 ± 1.73 vs. 44.671.74%, but were statistically the same as Cikta (40.8 ± 1.41%) during the breeding season. The Tsigai and Cikta spermatozoa had the same WOB of 53.89 ± 0.93 and 50.37 ± 0.98%, significantly (*p* < 0.001) higher than Rack 48.00 ± 0.80% during the breeding season. The Tsigai breed VCL was significantly higher (*p* < 0.001) during the out of season than the breeding season (197.70 ± 5.38 vs. 194.10 ± 10.90 μm/s), while LIN and WOB were higher during the breeding season than out of season (44.67 ± 1.74 vs. 40.27 ± 0.82% and 53.89 ± 0.93 vs. 49.84 ± 0.57%). 

The effects of ram on motility, viability, and chromatin condensation characteristics of Tsigai breed post-thaw spermatozoa based on the representative spermatozoa samples are presented in Supplementary Table S1. The spermatozoa standard motility parameters of the Tsigai breed were statistically the same among the rams. However, the VCL, VAP, LIN, STR, ALH, WOB, all intact spermatozoa, and the percentage of spermatozoa with IHITIA were significantly different (*p* < 0.05) among the rams. 

The effects of ram on motility, viability, and chromatin condensation characteristics of Cikta ram post-thaw spermatozoa based on the representative samples are presented in Supplementary Table S2. No significant difference (*p* > 0.05) exists among the Cikta rams in the standard motility parameters, VCL, VAP, VSL, BCF, WOB, percentage of spermatozoa categorized as IHITIA, and the Feulgen fragmentation. However, the LIN, STR, and ALH parameters and the percentage of all intact spermatozoa differed significantly among the Cikta rams. 

The effects of ram on motility, viability, and chromatin condensation characteristics of Racka breed post-thaw spermatozoa based on the representative spermatozoa samples are presented in Supplementary Table S3. There was no significant difference (*p* > 0.05) among the Racka rams in standard motility, VCL, VAP, STR, ALH, and Feulgen fragmentation parameters. Nevertheless, the LIN, BCF, WOB, percentage of all intact spermatozoa, and IHITIA categories significantly differed among the rams. 

Table 9 presents the effects of breed and season on spermatozoa morphometric parameters of the three native Hungarian breeds. The SD area of Racka ram spermatozoa was significantly (*p* < 0.05) higher than that of Cikta (1.04 ± 0.03 vs. 0.95 ± 0.03), but statistically the same as Tsigai rams (1.04 ± 0.03 vs. 0.97 ± 0.02 μm). The season does not affect all the parameters studied. However, average perimeter and sperm nucleus length presented significant breed and season interaction. 

The effects of breed and season interaction on the average perimeter and sperm nucleus length of the three native Hungarian ram spermatozoa are presented in Table 10. The average perimeter of Racka ram spermatozoa was significantly higher (*p* < 0.05) during the breeding season than out of season (18.46 ± 0.08 vs. 18.17 ± 0.08). The Cikta spermatozoa head was longer than that of Tsigai (7.06 ± 0.04 vs. 6.89 ± 0.03 μm) but statistically the same as that of Racka (6.97 ± 0.06 μm) during the breeding season.

The effects of Tsigai and Cikta rams on spermatozoa head morphometry are presented in Supplementary Tables S4 and S5, respectively, while those of Racka are presented in Supplementary Table S6. The results showed that the Tsigai and Cikta rams differed significantly (*p* < 0.005) on average area, average perimeter, and nucleus length parameters, but the standard deviations of all the parameters were statistically the same (*p* > 0.05). However, there was no significant (*p* > 0.05) difference in all the spermatozoa head morphometry parameters studied among the Racka rams.

## 4. Discussion

The Hungarian native sheep breeds, particularly Tsigai, Cikta, and Racka, are adaptable to the varied Hungarian weather conditions and serve significant roles in the livelihood of the commoners as a source of mutton, milk, wool, and landscape conservation of extensively cultivated pastures [5]. These valuable breeds face a severe extinction threat, ranging from endangered to vulnerable [16,17,18]. In recent years, there have been reports of an increase in the selection pressure globally, resulting in livestock initial gene pool fragmentation with a consequent loss of valuable native livestock genetic resources [9,29], with the extinction rate occurring at 1000–10,000-fold greater than the natural rate [30]. Despite the fact that some of these breeds were introduced to Hungary, they are now among Hungary’s heritage breeds, and they have contributed immensely to the livelihood of Hungarians, mainly rural dwellers. This calls for establishing Gene Banks or combining ex situ and in situ techniques to manage this devastating trend [31]. Gene Banks/cryo-banks are collections of biological samples kept for research or management [30]. Cryo-banking permits the indefinite conservation of valuable genetic resources as insurance for increasing the population of threatened breeds or regenerating extinct breeds back to the population [30]. Hence, the current study assessed the spermatozoa quality parameters of three native Hungarian sheep breeds collected and stored in the Gene Bank. 

The fresh ejaculate quality characteristics revealed that volume, spermatozoa concentrations, and subjective motility differ significantly among the breeds regardless of season. It agrees with the results of Barbas et al. [32]. The Tsigai produced a larger quantity of ejaculate but with lower sperm concentration; the Cikta and Racka produced less quantity but more concentrated semen. Racka had the highest spermatozoa concentration and subjective motility. Motility is one of the classical indicators of spermatozoa quality because high sperm motility was associated with a higher fertilization rate in different species [30]. Ejaculates collected during the breeding season had significantly higher volume and total spermatozoa number/ejaculate than those collected during the out of season. Our results supported the findings of Benia et al. [33] that ram ejaculate volume is highly influenced by season but contrasted the results of Barbas et al. [32]. The spermatozoa concentration and total sperm number/ejaculate presented significant breed and season interaction. This was possibly due to the higher concentration and total sperm number observed in Racka than in the other breeds. This was in agreement with the findings of Barbas et al. [32]. We observed that season had no significant effects on the earlier-mentioned parameters of the Cikta breed. This agrees with the findings of Gáspárdy et al. [2] that the Cikta breed is less seasonal among the Hungarian native sheep breeds. Moreover, we observed that the breeds differ more during the breeding season than the out of season. Semen samples of almost all studied Racka rams, except one animal, were collected in BS and OS. Based on the data of these ejaculates, the Racka breed produced significantly higher volume, sperm concentration, and total sperm number/ejaculate in BS than in OS. These findings agree with Sarlós et al.’s results [4]. The three breeds were the same in terms of the proportion of the fresh spermatozoa samples suitable for freezing based on 75% subjective motility and 2 billion/mL spermatozoa concentration threshold values, and post-thaw samples are ideal for storage in the Gene Bank based on 40% total motility. Our findings revealed that collecting the sample during the out of season resulted in a significantly higher proportion of ejaculates suitable for freezing. This could be because only half of the rams had in-season and out of season samples; the other had only in-season or out of season samples, since the rams were taken to the artificial insemination station at different times and stayed only for the semen collection period, so ejaculates of different rams were also evaluated in season and out of season.

It is essential to note that ram field fertility cannot be determined by only CASA motility parameters [34]. Interestingly, the Racka spermatozoa had better motility and progressive motility parameters than the Tsigai and Cikta breeds. Our results contradict those of Vozaf et al. [35], who did not find significant differences among three Slovak sheep breeds in most of the quality parameters of cryopreserved sperm; however, they analyzed electro-ejaculated spermatozoa and heterospermic samples. However, the velocity parameters (VCL, VAP, and VSL) were like those of Cikta but higher than those of Tsigai. Higher velocity is associated with increased cervical mucus penetration and fertilization rate [36]. In contrast, the spermatozoa of Tsigai and Cikta breeds had more linear movement, as indicated by their better LIN, STR, ALH, and WOB than those of the Racka breed. Average litter size had been reported to be strongly associated with LIN and STR, r = 0.87 and 0.77, respectively [37]. However, the motility and velocity parameters were not affected by season, while most of the trajectory parameters were better during the out of season than in the breeding season, which might be due to the reasons related to the collected samples highlighted earlier. Our findings on the effects of season on motility parameters contrast those of [38]. The disparity in the results might be because we used rams of different breeds. The results of the breed and season interaction effect on the VCL, LIN, and WOB further proved that the breeds differ more during the breeding season than out of season and that the spermatozoa of the Cikta breed are less seasonal than the Tsigai and Racka breeds. Moreover, from the representative ram samples, we observed that the motility parameters differed more among the breeds than within the breed, and most parameters were unaffected by season. Our previous study on ram epididymal spermatozoa revealed breed effects on motility parameters [39]. Additionally, the Racka breed spermatozoa moved faster but less linearly with higher ALH than the Tsigai’s and Cikta’s. The percentage of spermatozoa with intact plasma membranes and normal morphology and Feulgen fragmentation was not affected by breed. This contrasts with the report of Barbas et al. [32], which analyzed only head membrane integrity using eosin-nigrosine staining. We also observed that the viability, proportion of “live” morphological normal spermatozoa, and chromatin condensation parameters differed more within than among the breeds, that is, more in Tsigai than in Cikta and Racka. This agrees with the findings of Vozaf et al. [35], who state that breed does not influence the incidence of spermatozoa morphological abnormalities. 

Spermatozoa head morphometry is another essential aspect that affects the fertilization rate in different species, including bulls [40] and rams [41,42]. Similarly, studies revealed that cryopreservation reduces sperm head size [43]. We evaluated the average area, which shows how large the sperm nucleus is; the perimeter indicates the width and the length together with their corresponding standard deviations. Except for the SD area, all the parameters studied were statistically the same among the breeds and were unaffected by season. The breed and season interaction revealed that the sperm nucleus length differed more among the breeds during the breeding season. The Cikta spermatozoa had a longer head, although more comparable to the Racka than the Tsigai. The Tsiagi’s spermatozoa heads are longer during the out of season than in the breeding season. The fertility rate was reportedly strongly associated with the proportion of spermatozoa with short and elongated heads [42]. We observed differences in specific head morphometric traits among the rams of Tsigai and Cikta breeds but not in the Racka breed.

The program aimed to preserve genotypes representing outstanding genetic value, which did not allow the selection of individuals according to age and keeping conditions. The previous housing and feeding conditions of the animals may have had an influencing effect on individual semen quality, although the long quarantine period and the time spent in training for semen collection, which lasted at least 2–3 months, definitely reduced the possible differences in condition at the time of arrival, since, after this time, the housing and feeding system was the same. Differences between breeding farms appeared more in the rams and how they were accustomed to people and trained. The gene preservation project was not a classical experiment, so the rams only stayed at the station during quarantine, training, and sperm production. After collecting the appropriate doses, they were returned to the breeder. Thus, we collected samples from some rams only in season or, on the contrary, out of season, half of the rams in both periods. When planning a new program, if the budget allows, it is recommended to extend the sperm collection period for research purposes so that seasonal differences can be compared more profoundly.

During the program, the quality criteria of cryopreserved sperm were adjusted to commercial semen quality criteria when storing Gene Bank samples. In the future, these criteria will need to be reconsidered since, in the case of a gene preservation program, some important genotypes must be preserved even if the quality of the individual’s frozen sperm sample is lower. In the case of extensively kept rams, the biggest problem was getting used to sperm collection, in many cases failing to collect samples. Therefore, the use of the electro-ejaculator must be considered in the case of rams that cannot be trained to collect sperm, and in the case of the death of a ram of outstanding genetic value, the possibility of epididymal spermatozoa retrieval post-mortem should also be considered.

Following the evaluation of several spermatozoa traits presented in this study, further functional tests and the in vitro fertilization ability of the frozen spermatozoa are also intended to determine the suitability of the stored semen samples in the Gene Bank. For the maintenance of rare and valuable genotypes, cryo-conservation of not only spermatozoa but also oocytes and embryos is needed. Therefore, we plan to apply for support for an extended gene conservation program involving a more significant number of individuals of native Hungarian sheep breeds.

## 5. Conclusions

The current study observed individual and breed differences on several parameters of frozen/thawed spermatozoa; nonetheless, most of the frozen samples showed good quality with high motility and movement characteristics of spermatozoa, more than 40% of sperm with intact membranes, and very low abnormal chromatin condensation (less than 1% of the spermatozoa), and there was no significant difference between storage rate among breeds. Therefore, the applied freezing procedure is suitable for the implementation of further gene conservation programs. Nevertheless, considering the breed differences, further studies are needed to determine the reasons for the variances and improve the quality parameters by optimizing the freezing protocol. Additionally, the fertility of the stored samples in the Gene Bank needs to be evaluated via artificial inseminations. In the case of the Tsigai and Cikta breeds, no comprehensive comparative study has yet been conducted regarding the quality parameters of fresh and frozen semen samples. In the future, the quality criteria of frozen semen samples allowed to be stored in the Gene Bank will need to be reconsidered since, in the case of a gene preservation program, some critical genotypes must be preserved even if the quality of the individual’s frozen sperm sample is lower. The use of the electro-ejaculator must be considered in the case of rams that cannot be trained to collect sperm, and the possibility of post-mortem epididymal spermatozoa retrieval should also be considered in the case of a ram of outstanding genetic value. 

## Figures and Tables

**Table 1:** Number of animals and ejaculates collected, frozen and stored semen samples in the Gene Bank of each breed.

Breeds	Number of Rams	Number of Ejaculates Collected for Further Analysis and Processing	Number of Ejaculates Frozen	Number of Samples Frozen (Single, or Pooled of Two Ejaculates)	Number of Samples Stored in the Gene Bank
Tsigai	11	228	142	106	92
Cikta	7	135	80	65	60
Racka	6	98	63	48	47
Total	24	461	285	219	199

**Table 2:** Effects of breed and season on the fresh ejaculate’s quality characteristics of native Hungarian ram breeds (n = 24).

Parameters	Breed			*p*-Values	Season		*p*-Values	Breeds * Season *p*-Values
	Tsigai	Cikta	Racka		Breeding Season	Out of Season		
Volume (mL)	0.96 ± 0.02 ^a^	0.70 ± 0.03 ^b^	0.79 ± 0.04 ^b^	0.0001	0.91 ± 0.03 ^a^	0.79 ± 0.02 ^b^	0.0001	0.053
Concentration (×10^6^/mL)	3624.31 ± 131.20 ^a^	4707.19 ± 181.59 ^b^	5303.31 ± 155.83 ^c^	0.0001	4322.00 ± 133.7	4265.01 ± 135.33	0.257	0.0001
Total sperm number/ejaculate (×10^6^/mL)	3630.50 ± 194.33	3434.25 ± 213.39	4485.06 ± 358.69	0.141	3966.80 ± 198.38 ^a^	3564.10 ± 195.91 ^b^	0.010	0.011
Mass movement (%)	3.75 ± 0.10	3.66 ± 0.14	3.59 ± 0.17	0.658	3.86 ± 0.10	3.51 ± 0.12	0.072	0.677
Subjective motility (%)	71.43 ± 0.94 ^a^	70.49 ± 1.30 ^a^	74.19 ± 0.96 ^b^	0.034	71.74 ± 080	71.79 ± 0.97	0.918	0.215

Means in the same row with different superscripts ^a,b,c^ differ significantly. Breed * Season: breed and season interaction.

**Table 3:** Breed and season interaction effects on the quality characteristics of native Hungarian fresh ram ejaculates (n = 24).

Parameters	Seasons	Breed			*p*-Values
		Tsigai	Cikta	Racka	
Concentration (10^6^/mL)	Breeding season	3191.38 ± 174.24 ^Aa^	4662.78 ± 260.93 ^b^	5630.79 ± 178.57 ^Ac^	0.0001
	Out of season	3937.81 ± 183.70 ^Ba^	4750.33 ± 254.57 ^b^	4639.24 ± 273.97 ^Bab^	0.010
*p*-values		0.0001	0.705	0.011	
Total sperm number/ejaculate	Breeding season	3478.92 ± 238.69 ^a^	3411.79 ± 278.30 ^a^	5185.53 ± 485.88 ^Ab^	0.0001
	Out of season	3740.25 ± 287.08	3456.06 ± 324.28	3064.66 ± 362.22 ^B^	0.423
*p*-values		0.899	0.872	0.0001	

Means in the same column ^A,B^ or row ^a,b,c^ with different superscripts differ significantly.

**Table 4:** Effects of breed and season on the proportion of the fresh spermatozoa samples suitable for freezing based on 75% subjective motility and 2 billion/mL spermatozoa concentration threshold values and post-thaw samples ideal for storage in the Gene Bank based on 40% total motility of the three native Hungarian sheep breeds (n = 24).

Parameters		Breed		Chi-Square Value	Season		Chi-Square Value
	Tsigai	Cikta	Racka		Breeding Season	Out of Season	
Proportion of ejaculates suitable for freezing (%)	62.70	58.20	64.30	0.586	56.00 ^a^	66.80 ^b^	0.017
Storage rate (%)	86.80	92.30	97.90	0.076	88.70	92.60	0.312

Means in the same row with different superscripts ^a,b^ differ significantly.

**Table 5:** Effects of breed and season on the freezability/post-thaw sperm motility and kinematic parameters of some native Hungarian sheep breeds (n = 24).

Parameters	Breed			*p*-Values	Season		*p*-Values	*p*-Value Breed * Season
	Tsigai	Cikta	Racka		Breeding Season	Out of Season		
Total motility (%)	60.55 ± 1.1 ^a^	57.33 ± 1.4 ^b^	70.20 ± 1.5 ^c^	0.0001	62.54 ± 1.1	62.85 ± 1.1	0.844	0.068
Progressive motility (%)	53.92 ± 1.2 ^a^	51.24 ± 1.5 ^a^	65.99 ± 1.7 ^b^	0.0001	56.16 ± 1.2	57.94 ± 1.2	0.296	0.100
Curvilinear velocity (μm/s)	188.61 ± 2.7 ^a^	203.08 ± 3.3 ^b^	209.62 ± 3.7 ^b^	0.0001	197.99 ± 2.7	202.88 ± 2.6	0.197	0.011
Average path velocity (μm/s)	95.71 ± 1.2 ^a^	102.18 ± 1.4 ^b^	102.77 ± 1.6 ^b^	0.0001	99.37 ± 1.1	101.07 ± 1.1	0.287	0.085
Straight line velocity (μm/s)	76.64 ± 1.0 ^a^	82.59 ± 1.2 ^b^	80.63 ± 1.3 ^b^	0.0001	78.69 ± 1.0	81.22 ± 0.9	0.065	0.204
Linearity (%)	40.48 ± 0.4 ^a^	40.45 ± 0.5 ^a^	37.98 ± 0.5 ^b^	0.0001	39.40 ± 0.4	39.87 ± 0.4	0.382	0.027
Straightness (%)	79.34 ± 0.7 ^a^	80.63 ± 0.9 ^a^	76.54 ± 1.0 ^b^	0.008	77.49 ± 0.7 ^a^	80.18 ± 0.7 ^b^	0.008	0.321
Beat cross frequency (Hz)	31.42 ± 0.3	32.02 ± 0.3	31.17 ± 0.4	0.191	31.03 ± 0.3 ^a^	32.04 ± 0.3 ^b^	0.009	0.860
Amplitude of lateral head displacement (μm)	5.50 ± 0.1 ^a^	5.65 ± 0.1 ^a^	6.01 ± 0.1 ^b^	0.0001	5.84 ± 0.1 ^a^	5.60 ± 0.1 ^b^	0.019	0.129
Wobble (%)	50.72 ± 0.4 ^a^	49.85 ± 0.5 ^a^	47.64 ± 0.6 ^b^	0.0001	49.68 ± 0.4	49.13 ± 0.4	0.361	0.004

Means in the same row with different superscripts ^a,b,c^ differ significantly. Breed * Season: breed and season interaction.

**Table 6:** Breed and season interaction effects on post-thaw sperm kinematic parameters of native Hungarian sheep breeds (n = 24).

Parameters	Seasons	Breeds			*p*-Values
		Tsigai	Cikta	Racka	
Curvilinear velocity (μm/s)	Breeding season	178.70 ± 3.6 ^Aa^	203.88 ± 4.0 ^b^	211.39 ± 3.9 ^b^	0.0001
	Out of season	198.53 ± 4.0 ^B^	202.29 ± 4.5	207.84 ± 4.3	0.161
*p*-value		0.0001	0.809	0.635	
Linearity (%)	Breeding season	41.12 ± 0.8 ^a^	40.04 ± 0.6 ^a^	37.00 ± 0.5 ^Ab^	0.0001
	Out of season	39.90 ± 0.5	40.69 ± 0.8	38.79 ± 0.5 ^B^	0.174
*p*-value		0.126	0.798	0.027	
Wobble (%)	Breeding season	52.30 ± 0.7 ^Aa^	49.70 ± 0.8 ^b^	47.04 ± 0.8 ^c^	0.001
	Out of season	49.15 ± 0.5 ^B^	50.00 ± 0.7	48.25 ± 0.9	0.125
*p*-value		0.0001	0.776	0.307	

Means in the same column ^A,B^ or row ^a,b,c^ with different superscripts differ significantly.

**Table 7:** Effects of breed and season on sperm quality parameters of representative post-thaw sperm samples of some native Hungarian ram breeds (n = 24).

Parameters	Breed			*p*-Values	Season		*p*-Values	*p*-Value Breed * Season
	Tsigai	Cikta	Racka		Breeding Season	Out of Season		
Total motility (%)	62.71 ± 1.73 ^a^	62.86 ± 2.66 ^a^	72.26 ± 2.03 ^b^	0.004	65.59 ± 1.89	67.09 ± 1.69	0.555	0.208
Progressive motility (%)	55.82 ± 2.07 ^a^	57.29 ± 2.53 ^a^	67.11 ± 2.26 ^b^	0.002	59.17 ± 2.15	61.27 ± 1.94	0.470	0.454
Curvilinear velocity (μm/s)	186.30 ± 4.41 ^a^	205.67 ± 5.73 ^b^	205.79 ± 5.29 ^b^	0.001	196.47 ± 4.58	197.57 ± 4.15	0.858	0.039
Average path velocity (μm/s)	94.98 ± 1.78 ^a^	104.11 ± 2.11 ^b^	100.46 ± 2.59 ^ab^	0.005	100.11 ± 1.96	98.66 ± 1.77	0.584	0.283
Straight line velocity (μm/s)	76.76 ± 1.41 ^a^	84.06 ± 2.06 ^b^	78.49 ± 2.60 ^ab^	0.030	80.27 ± 1.80	79.02 ± 1.63	0.608	0.835
Linearity (%)	41.15 ± 0.75 ^a^	40.66 ± 1.08 ^a^	37.26 ± 0.64 ^b^	0.001	40.63 ± 0.75	39.88 ± 0.68	0.461	0.037
Straightness (%)	77.99 ± 2.53	80.14 ± 1.07	76.95 ± 0.74	0.079	77.73 ± 1.86	79.51 ± 1.69	0.481	0.354
Beat cross frequency (Hz)	31.64 ± 0.47	32.11 ± 0.66	30.64 ± 0.69	0.301	31.57 ± 0.55	31.51 ± 0.50	0.932	0.617
ALH (μm)	5.39 ± 0.13 ^a^	5.68 ± 0.17 ^ab^	6.03 ± 0.09 ^b^	0.013	5.78 ± 0.11	5.58 ± 0.09	0.180	0.070
Wobble (%)	50.73 ± 0.53 ^a^	50.33 ± 0.77 ^ab^	48.26 ± 0.51 ^b^	0.002	50.79 ± 0.53	49.66 ± 0.47	0.115	0.024
All intact sperm (%)	44.94 ± 1.71	43.47 ± 2.11	46.04 ± 2.65	0.864	45.96 ± 2.0	43.40 ± 1.8	0.335	0.733
IHITIA (%)	43.19 ± 1.79	41.86 ± 2.17	44.92 ± 2.58	0.845	45.04 ± 2.0	41.58 ± 1.8	0.201	0.805
Abnormal chromatin condensation (%)	0.06 ± 0.04	0.12 ± 0.07	0.00 ± 0.00	0.171	0.06 ± 0.04	0.07 ± 0.03	0.841	0.248

Means in the same row with different superscripts ^a,b^ differ significantly. IHITIA: intact head, intact tail, intact acrosome, normal morphology; all intact sperm: spermatozoa with intact membranes. Breed * Season: breed and season interaction.

**Table 8:** Breed and season interaction effects on some kinematic parameters of representative frozen/thawed sperm samples of the three native Hungarian sheep breeds (n = 24).

Parameters	Seasons	Breeds			*p*-Values
		Tsigai	Cikta	Racka	
Curvilinear velocity (μm/s)	Breeding season	194.50 ± 10.90 ^Aa^	202.10 ± 11.70 ^b^	200.60 ± 9.78 ^b^	0.0001
	Out of season	197.70 ± 5.38 ^B^	200.09 ± 9.11	200.50.14.15	0.434
*p*-value		0.025	0.802	0.179	
Linearity (%)	Breeding season	44.67 ± 1.74 ^Aa^	40.8 ± 1.41 ^ab^	39.00 ± 1.73 ^b^	0.0001
	Out of season	40.27 ± 0.82 ^B^	39.00 ± 1.67	39.00 ± 1.51	0.826
*p*-value		0.009	0.945	0.286	
Wobble (%)	Breeding season	53.89 ± 0.93 ^Aa^	50.37 ± 0.98 ^a^	48.00 ± 0.80 ^b^	0.0001
	Out of season	49.84 ± 0.57 ^B^	50.31 ± 1.22	48.71 ± 0.81	0.959
*p*-value		0.001	0.661	0.512	

Means in the same column ^A,B^ or row ^a,b^ with different superscripts differ significantly.

**Table 9:** Effects of breed and season on spermatozoa morphometric parameters of some native Hungarian rams (n = 24).

Parameters	Breeds			*p*-Values	Seasons		*p*-Values	Interaction *p*-Values
	Racka	Tsigai	Cikta		BS	OS		
Average area (μm)	20.53 ± 0.14	20.43 ± 0.11	20.48 ± 0.12	0.695	20.58 ± 0.11	20.39 ± 0.09	0.122	0.099
SD Area (μm)	1.04 ± 0.03 ^a^	0.97 ± 0.02 ^ab^	0.95 ± 0.03 ^b^	0.014	0.97 ± 0.02	0.99 ± 0.19	0.342	0.504
Average perimeter (μm)	18.22 ± 0.06	18.30 ± 0.05	18.33 ± 0.05	0.670	18.38 ± 0.05	18.28 ± 0.04	0.055	0.039
SD Perimeter (μm)	0.64 ± 0.05	0.85 ± 0.13	0.72 ± 0.11	0.134	0.77 ± 0.13	0.74 ± 0.07	0.214	0.086
Head length (μm)	6.95 ± 0.04	6.99 ± 0.02	7.03 ± 0.03	0.134	6.97 ± 0.03	7.01 ± 0.02	0.762	0.023
SD Head length (μm)	0.26 ± 0.02	0.28 ± 0.01	0.31 ± 0.04	0.134	0.25 ± 0.01	0.30 ± 0.02	0.214	0.086

Means in the same row ^a,b^ with different superscripts differ significantly.

**Table 10:** Breed and season interaction effects on the average perimeter and sperm nucleus length of some native Hungarian rams’ spermatozoa (n = 24).

Parameters	Season		Breeds		*p*-Values
		Racka	Tsigai	Cikta	
Average perimeter	Breeding season	18.46 ± 0.08 ^A^	18.24 ± 0.09	18.42 ± 0.05	0.131
	Out of season	18.17 ± 0.08 ^B^	18.32 ± 0.05	18.27 ± 0.07	0.427
*p*-values		0.015	0.363	0.146	
Nucleus length (μm)	Breeding season	6.97 ± 0.06 ^ab^	6.89 ± 0.03 ^Aa^	7.06 ± 0.04 ^b^	0.045
	Out of season	6.91 ± 0.03	7.04 ± 0.27 ^B^	7.00 ± 0.04	0.157
*p*-values		0.428	0.010	0.347	

Means in the same column ^A,B^ or row ^a,b^ with different superscripts differ significantly.

## Data Availability

The data presented in this study are available upon request from the corresponding author.

## Supplementary Materials

The following supporting information can be downloaded at: https://www.mdpi.com/article/10.3390/vetsci11080337/s1, Table S1: Effects of RAM on motility, viability and chromatin condensation characteristics of Cigája breed post-thaw spermatozoa based on the representative sperm samples (n = 11), Table S2: Effects of RAM on motility, viability and chromatin condensation characteristics of Cikta breed post-thaw spermatozoa based on the representative sperm samples (n = 7), Table S3: Effects of RAM on motility, viability and chromatin condensation characteristics of Racka breed post-thaw spermatozoa based on the representative sperm samples (n = 6), Table S4: Effects of representative rams on spermatozoa morphometric parameters of Cigája rams (n = 24), Table S5: Effects of representative rams on spermatozoa morphometric parameters of Cikta rams (n = 24), Table S6: Effects of representative rams on spermatozoa morphometric parameters of Racka rams (n = 24).
