# Supplementary material for: In Vitro Gene Conservation Status and the Quality of the Genetic Resources of Native Hungarian Sheep Breeds
Source: Vet Sci. 2024 Jul 25;11(8):337. doi: 10.3390/vetsci11080337 (PMC11360158; doi:10.3390/vetsci11080337)
Supplement: Supplementary file 1 [file vetsci-11-00337-s001.zip › vetsci-3091402-supplementary.pdf]

Supplementary tables

Table S1. Effects of RAM on motility, viability and chromatin condensation characteristics of Tsigai breed post-thaw spermatozoa based on the representative sperm samples (n=11)

Parameters	Rams											p-values
	96	165	311	321	466	912	3241	4109	5152	5196	5210	
Total motility (%)	67.33±2.2	51.00±3.8	62.67±2.7	73.67±5.2	56.67±2.8	73.33±4.6	61.33±5.4	56.33±7.9	62.83±0.2	62.67±10.2	61.00±5.1	**0.076**
Progressive motility (%)	59.67±4.1	44.33±3.8	57.67±4.3	71.00±5.1	44.33±5.1	68.00±5.5	58.67±4.4	50.67±8.5	54.00±2.9	55.00±10.9	50.67±6.4	**0.080**
Curvilinear velocity (µm/s)	210.33±12.4^{a} ^{c}	181.33±7.2 ^{ab}	181.33±5.8^{a} ^{b}	200.33±6.4^{a}	166.67±10.4	225.00±3.6^{c}	215.67±7.3^{c}	169.00±11.1 ^{b}	149.33±6.4	177.67±3.4^{a} ^{b}	172.67±6.6 ^{ab}	**0.0001**
Average path velocity (µm/s)	105.53±5.5 ^{ac}	91.80±1.2^{abc}	88.87±1.8 ^{ab}	99.70±2.5^{ab} ^{c}	86.90±5.7^{a}	109.00±1.5 ^{c}	105.00±2.5^{a}	87.13±6.4^{a}	83.77±6.0 ^{b}	95.60±0.9^{abc}	91.47±4.9^{abc}	**0.002**
Straight line velocity (µm/s)	85.23±4.6	77.83±1.2	68.63±0.9	76.43±2.3	73.23±4.8	87.17±1.7	78.33±2.6	71.70±6.1	73.07±7.6	78.60±3.7	74.17±4.7	**0.126**
Linearity (%)	40.72±0.5^{ab} ^{c}	42.80±2.2 ^{ab}	37.89±1.9 ^{a}	38.29±0.8 ^{a}	44.17±0.4^{a} ^{b}	39.25±1.2^{a}	35.95±1.0 ^{a}	42.57±1.0^{a} ^{bc}	47.26±2.6^{b} ^{c}	43.59±2.1^{abc}	42.97±2.2^{abc}	**0.003**
Straightness (%)	80.33±0.3^{ab} ^{c}	84.33±2.3 ^{a}	77.00±1.5^{c}	76.33±0.7 ^{c}	84.75±0.6^{a} ^{b}	79.33±1.5^{ab}	73.67±0.9 ^{c}	82.00±1.0^{a} ^{bc}	86.00±3.1^{a} ^{b}	81.33±2.9^{abc}	80.67±0.9^{abc}	**0.005**
Beat cross frequency (Hz)	33.27±0.5	32.63±1.2	30.07±0.7	31.07±0.5	31.50±0.6	32.73±1.0	29.67±0.7	30.10±1.4	34.97±3.2	32.33±1.9	29.67±1.8	**0.264**
Amplitude of lateral head displacement (µm)	5.59±0.1^{c}	4.72±0.4^{c}	5.76±0.3^{bc}	5.84±0.1^{bc}	4.79±0.3^{ac}	6.06±0.2^{b}	6.58±0.2^{b}	5.15±0.1^{a}	4.17±0.5^{a}	5.35±0.3^{a}	5.38±0.1^{ab}	**0.0001**

Wobble (%)	49.96±0.8^{ab}	50.39±1.2^{ab}	48.84±1.2^{a}	49.67±0.8^{a}	52.04±0.4^{ab}	47.83±0.8^{a}	48.27±0.7^{a}	51.33±0.6^{a}	55.19±1.5^{b}	53.45±1.0^{b}	52.43±2.0^{ab}	**0.002**
All intact sperm (%)	49.67±4.8^{a}	46.83±9.1^{ab}	44.06±2.9^{ab}	53.60±2.1^{a}	27.53±4.0^{b}	49.77±1.8^{a}	50.77±1.6^{a}	33.30±2.9^{b}	44.40±1.9^{ab}	53.83±3.7^{a}	40.57±1.7^{ab}	**0.002**
IHITIA (%)	49.17±4.6^{a}	46.33±9.2^{a}	41.01±3.0^{ab}	52.00±2.3^{a}	24.37±4.5^{b}	48.67±1.4^{a}	49.00±0.9^{a}	31.63±1.9^{b}	39.83±1.9^{ab}	53.50±3.4^{a}	39.57±1.4^{ab}	**0.0001**
Feulgen Fragmentation (%)	0.00±0.0	0.00±0.0	0.00±0.0	0.17±0.17	0.00±0.0	0.00±0.0	0.33±0.33	0.17±0.17	0.00±0.0	0.00±0.0	0.00±0.0	**0.625**

Means in the same row with different superscripts^{a,b,c} differ significantly.

Table S2. Effects of RAM on motility, viability and chromatin condensation characteristics of Cikta breed post-thaw spermatozoa based on the representative sperm samples (n=7)

**Parameters**	Rams							**p-value**
	246	366	395	410	560	2283	3122	
Total motility (%)	67.00±4.05	69.67±5.76	62.33±2.40	70.67±2.34	49.33±8.36	51.67±10.69	69.33±4.64	**0.186**
Progressive motility (%)	61.67±5.21	64.67±3.84	56.00±2.89	64.67±0.33	44.33±7.97	47.67±10.39	62.00±4.93	**0.136**
Curvilinear velocity	217.67±23.02	210.00±10.21	215.00±13.00	186.67±3.33	170.67±1.76	215.67±9.74	224.00±15.89	**0.096**
Average path velocity	105.63±7.77	109.33±6.36	102.23±5.39	98.77±3.36	94.50±3.40	106.00±1.53	112.33±6.57	**0.309**
Straight line velocity	79.83±6.23	90.07±7.02	76.43±5.74	80.80±5.69	82.00±4.41	88.47±1.12	90.83±5.08	**0.399**
Linearity (%)	36.73±1.66[a]	42.94±1.68[ab]	34.16±1.97[a]	42.19±3.22[ab]	47.30±1.89[b]	41.10±1.21[ab]	40.93±1.35[ab]	**0.007**
Straightness (%)	75.00±1.00[ab]	81.67±1.80[ab]	73.67±2.23[a]	81.00±3.10[ab]	86.00±2.00[b]	83.33±1.20[b]	80.33±0.88[ab]	**0.005**
Beat cross frequency	29.03±0.15	32.67±2.37	29.17±1.80	32.90±1.73	35.17±1.6	32.53±0.95	33.27±0.22	**0.097**
Amplitude of lateral head displacement (μm)	6.34±0.16[a]	5.86±0.20[a]	6.35±0.17[a]	5.17±0.34[b]	4.38±0.44[b]	5.50±0.19[ab]	6.16±0.32[a]	**0.004**
Wobble (%)	48.76±1.64	52.29±1.41	46.92±1.51	51.63±2.46	54.74±1.54	48.59±2.2	50.09±1.23	**0.098**
All intact sperm (%)	55.60±3.58[a]	44.89±1.73[ab]	33.33±1.59[bc]	47.17±5.19[ac]	34.00±6.71[bc]	43.67±4.91[ac]	45.50±4.25[ac]	**0.036**
IHITIA (%)	54.27±4.18	44.10±1.73	32.90±1.25	44.33±5.53	31.67±8.37	41.80±3.82	44.00±4.95	**0.059**
Feulgen Fragmentation (%)	0.33±0.33	0.00±0.00	0.00±0.00	0.00±0.00	0.33±0.33	0.00±0.00	0.17±0.17	**0.672**

Means in the same row with different superscripts [a,b,c] differ significantly.

Table S3. Effects of RAM on motility, viability and chromatin condensation characteristics of Racka breed post-thaw spermatozoa based on the representative sperm samples (n=6)

**Parameters**	**Rams**						**p-value**
	32	38	310	483	1188	1256	
Total motility (%)	67.33±9.34	76.00 ±4.00	73.67±4.48	68.00±4.10	67.00±1.15	76.00±5.13	**0.898**
Progressive motility (%)	56.33±7.69	73.33±4.70	67.33±5.69	68.33±4.26	63.67±0.88	72.00±5.35	**0.298**
Curvilinear velocity (μm/s)	195.00±13.74	222.00±9.64	200.00±2.52	200.33±21.83	255.00±10.26	195.75±11.26	**0.409**
Average path velocity (μm/s)	93.03±4.07	114.33±6.49	97.20±1.22	96.27±9.54	104.90±4.68	97.90±5.40	**0.202**
Straight line velocity (μm/s)	71.60±4.01	93.53±6.83	76.90±2.14	73.00±7.67	82.23±7.04	74.88±4.53	**0.141**
Linearity (%)	36.43±2.26[ab]	41.83±1.04[a]	37.89±1.94[ab]	36.13±0.00[b]	33.65±1.27[b]	38.08±1.46[ab]	**0.049**
Straightness (%)	76.67±1.77	81.00±1.15	79.00±1.53	75.33±0.33	74.33±2.03	75.75±1.44	**0.067**
Beat cross frequency (Hz)	28.57±0.99[a]	35.57±2.04[b]	30.13±1.03[ab]	29.43±0.92[ab]	30.65±1.21[ab]	30.64±0.69[ab]	**0.026**
Amplitude of lateral head displacement (μm)	6.12±0.10	5.51±0.15	5.99±0.05	6.15±0.21	6.50±0.30	5.95±0.30	**0.275**
Wobble (%)	47.25±2.17[ab]	51.47±0.99[b]	47.65±1.40[ab]	47.89±0.45[ab]	45.62±0.00[a]	49.84±0.61[ab]	**0.037**
All intact sperm (%)	47.40±3.33[ab]	44.23±5.02[ab]	48.83±5.86[ab]	30.83±2.37[b]	41.80±2.36[ab]	58.88±6.18[a]	**0.021**
IHITIA (%)	46.40±3.26[ab]	43.80±4.93[ab]	46.37±5.63[ab]	29.87±2.24[b]	41.26±2.62[ab]	57.63±5.91[a]	**0.020**
Feulgen Fragmentation (%)	0.00±0.00	0.00±0.00	0.00±0.00	0.00±0.00	0.00±0.00	0.00±0.00	**0.705**

Means in the same row with different superscripts [a,b] differ significantly.

Table S4. Effects of representative rams on spermatozoa morphometric parameters of Tsigai rams (n=24)

Parameters	Rams											p-values
	96	165	311	321	466	912	3241	4109	5152	5196	5210	
Average area	20.79±0.29^{ac}	20.79±0.27^{a}	20.21±0.09^{ab}	20.30±0.17^{ab}	21.58±0.42^{c}	19.90±0.06^{ab}	20.44±0.17^{abc}	19.87±0.19^{ab}	19.55±0.24^{b}	20.63±0.28^{abc}	20.62±0.05^{abc}	**0.0001**
SD Area	0.98±0.08	1.06±0.05	1.00±0.07	1.01±0.12	0.97±0.03	1.02±0.06	0.99±0.15	0.90±0.04	0.88±0.02	0.96±0.02	0.89±0.08	**0.836**
Average perimetric	18.50±0.11^{ac}	18.35±0.09^{abc}	17.95±0.04^{b}	18.23±0.05^{abc}	18.72±0.19^{c}	18.10±0.03^{ab}	18.43±0.09^{abc}	18.26±0.07^{abc}	17.59±0.09^{b}	18.41±0.11^{abc}	18.42±0.07^{abc}	**0.0001**
SD Perimetric	0.70±0.19	0.58±0.07	0.60±0.04	0.52±0.07	0.45±0.02	0.50±0.02	1.08±0.55	1.28±0.38	0.80±0.34	1.08±0.39	1.72±1.26	**0.670**
Head length (um)	7.09±0.02^{ab}	6.94±0.06^{ab}	6.84±0.05^{a}	7.01±0.04^{ab}	7.21±0.09^{b}	7.01±0.06^{a}	7.13±0.07^{b}	7.02±0.04^{ab}	6.86±0.08^{a}	6.96±0.04^{ab}	6.88±0.05^{a}	**0.002**
SD Head length (um)	0.26±0.01	0.30±0.01	0.27±0.04	0.29±0.04	0.27±0.02	0.29±0.02	0.31±0.03	0.32±0.03	0.26±0.01	0.27±0.00	0.24±0.02	**0.628**

Means in the same row with different superscripts ^{a,b,c} differ significantly.

Table S5. Effects of representative rams on spermatozoa morphometric parameters of Cikta rams (n=24)

Parameters						Rams		**p-values**
	246	366	395	410	560	2283	3122	
Average area	20.74±0.07[a]	21.08±0.28[a]	20.51±0.21[ab]	20.67±0.27[a]	19.65±0.17[b]	20.10±0.25[ab]	20.28±0.08[ab]	**0.005**
SD Area	0.89±0.04	1.01±0.06	1.12±0.12	0.94±0.03	0.95±0.01	0.85±0.02	0.87±0.03	**0.065**
Average perimetric	18.47±0.03[a]	18.54±0.08[a]	18.40±0.07[a]	18.37±0.13[a]	17.89±0.06[b]	18.27±0.05[a]	18.37±0.04[a]	**0.0001**
SD Perimetric	0.81±0.20	0.95±0.49	0.90±0.43	0.46±0.01	0.53±0.06	0.91±049	0.45±0.04	**0.801**
Head length (um)	7.04±0.02[ab]	7.11±0.07[a]	6.96±0.01[ab]	7.16±0.09[a]	6.77±0.01[b]	7.12±0.04[a]	7.02±0.09[ab]	**0.006**
SD Head length (um)	0.27±0.03	0.28±0.03	0.24±0.03	0.60±0.28	0.24±0.00	0.26±0.01	0.25±0.03	**0.254**

Means in the same row with different superscripts [a,b] differ significantly.

Table S6. Effects of representative rams on spermatozoa morphometric parameters of Racka rams (n=24)

Parameters				Rams			**P-values**
	32	38	310	483	1188	1256	
Average area	20.60±0.06	19.77±0.21	20.51±0.29	20.87±0.32	20.54±0.44	20.80±0.38	**0.266**
SD Area	1.03±0.09	0.92±0.04	1.19±0.04	1.09±0.05	1.03±0.12	1.01±0.02	**0.188**
Average perimetric	18.41±0.04	18.02±0.14	18.38±0.12	18.54±0.15	18.41±0.19	18.37±0.16	**0.323**
SD Perimetric	0.81±0.22	0.58±0.12	0.69±0.13	0.52±0.03	0.60±0.06	0.64±0.13	**0.183**
Head length (um)	6.96±0.03	6.84±0.07	6.95±0.06	7.09±0.15	7.09±0.08	6.81±0.08	**0.441**
SD Head length (um)	0.32±0.09	0.26±0.03	0.24±0.02	0.271±0.04	0.233±0.04	0.242±0.04	**0.764**
